# Author Correction: RNA cytosine methylation and methyltransferases mediate chromatin organization and 5-azacytidine response and resistance in leukaemia

**DOI:** 10.1038/s41467-018-04518-9

**Published:** 2018-06-06

**Authors:** Jason X. Cheng, Li Chen, Yuan Li, Adam Cloe, Ming Yue, Jiangbo Wei, Kenneth A. Watanabe, Jamile M. Shammo, John Anastasi, Qingxi J. Shen, Richard A. Larson, Chuan He, Michelle M. Le Beau, James W. Vardiman

**Affiliations:** 1 Department of Pathology, University of Chicago, Chicago, IL 60637 USA; 2 University of Chicago Comprehensive Cancer Center, Chicago, IL 60637 USA; 3 Department of Haematology, Peking University First Hospital, Beijing, 100034 China; 4 Department of Chemistry, University of Chicago, Chicago, IL 60637 USA; 5 Genomics Core, Emory University, Atlanta, GA 30322 USA; 6 Rush University Medical Center, Chicago, IL 60612 USA; 7 University of Nevada, Las Vegas, NV 89154 USA; 8 Department of Medicine, University of Chicago, Chicago, IL 60637 USA

Correction to: *Nature Communications* 10.1038/s41467-018-03513-4, published online 21 March 2018

In the originally published version of this Article, the GAPDH loading control blot in Fig. 1a was inadvertently replaced with a duplicate of the DNMT2 blot in the same panel during assembly of the figure. This has now been corrected in both the PDF and HTML versions of the Article.

**Fig. 1:**
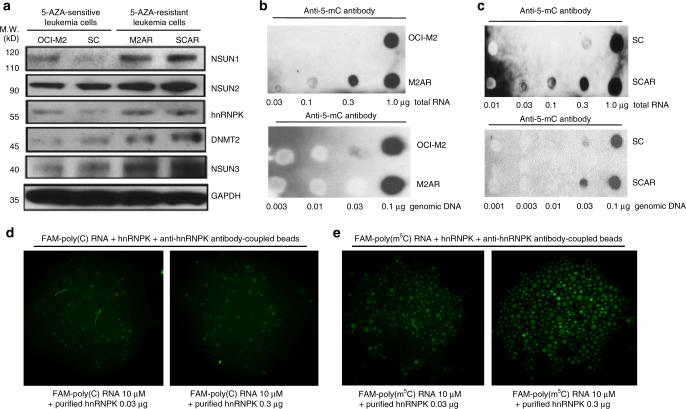
Differential expression of RNA:m^5^C, RCMTs and hnRNPK in 5-AZA-sensitive and 5-AZA-resistant leukaemia cells and the binding of hnRNPK to unmethylated and cytosine-methylated RNA. **a** Western blot analysis of expression of RCMTs, hnRNPK and other proteins in the 5-AZA-sensitive OCI-M2 and SC leukaemia cells and the 5-AZA-resistant M2AR and SCAR leukaemia cells. **b** Dot blot analysis of 5-methylcytosine (m^5^C) in RNA and DNA from OCI-M2 and M2AR cells. **c** Dot blot analysis of 5-methylcytosine (m^5^C) in RNA and DNA from SC and SCAR cells. **d**, **e** Visualization and measurement of the binding of purified recombinant hnRNPK to the unmethylated and cytosine-methylated fluorescein (FAM)-labelled RNA oligos by an antibody-coupled bead assay

