# Supplementary material for: RNA cytosine methylation and methyltransferases mediate chromatin organization and 5-azacytidine response and resistance in leukaemia
Source: Nat Commun. 2018 Mar 21;9:1163. doi: 10.1038/s41467-018-03513-4 (PMC5862959; doi:10.1038/s41467-018-03513-4)
Supplement: Supplementary file 1 — Supplementary Information [file 41467_2018_3513_MOESM1_ESM.pdf]

SUPPLEMENTARY FIGURES

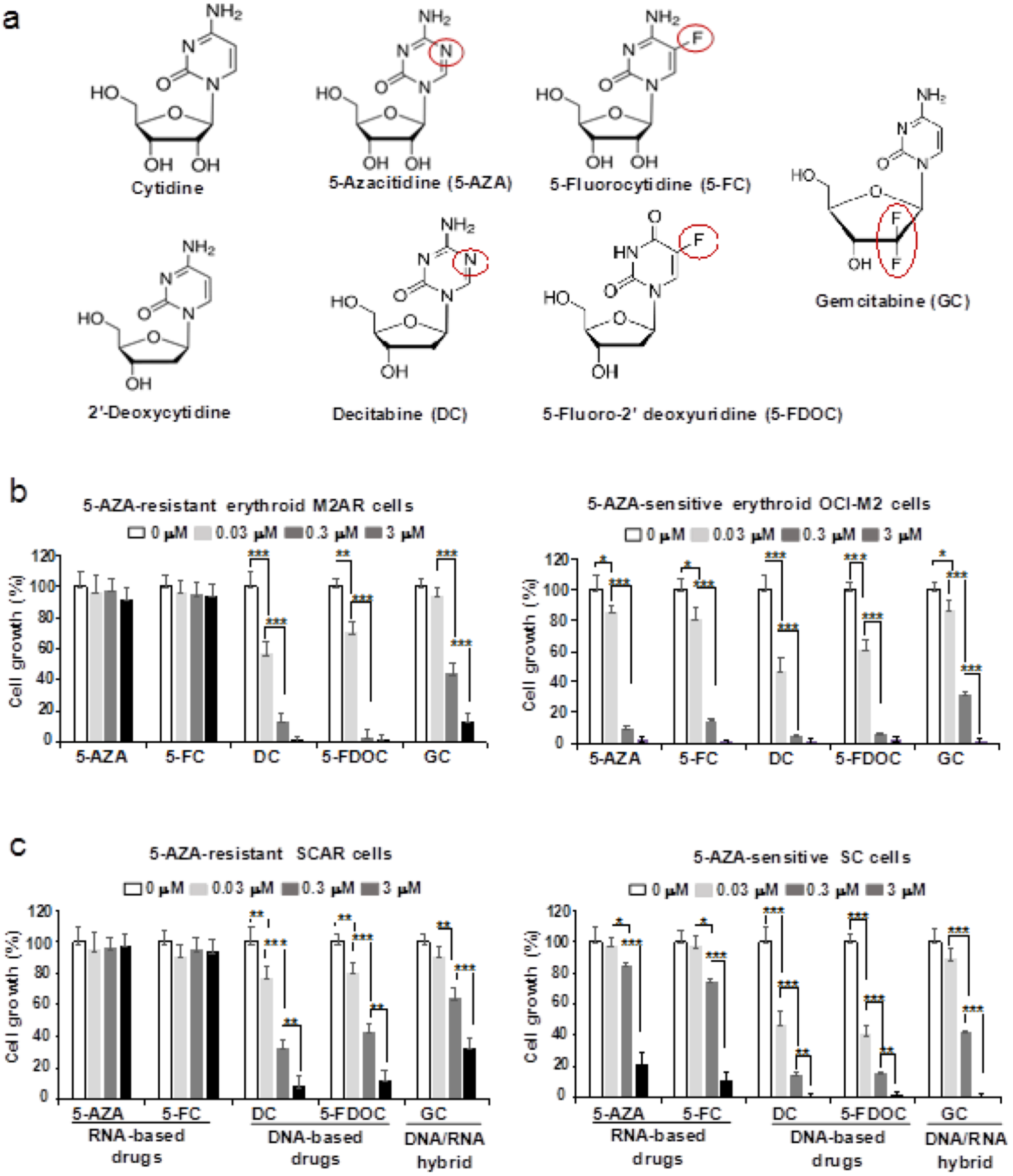

Supplementary Figure 1. The backbone chemistries of nucleic acid analogues/drugs and their effects on the response/resistance in leukeakemia cells. (a) Chemical structures of nucleic acid analogues used in this study. **(b)** Measurements of the effects of various nucleic acid analogues on cell growth by MTT assay in the 5-AZA-rsensitive and 5-resistant erythroid leukeakemia cell lines, OCI-M2 and M2AR. **(c)** Measurements of the effects of various nucleic acid analogues on cell growth by MTT assay in the 5-AZA-sensitive and 5-resistant monocytic leukeakemia cell lines, SC and SCAR.

Data are represented as the mean ± SEM of n = 3 independent samples. **P* < 0.05, ***P* < 0.01 and ****P* < 0.001 by Student's t-test.

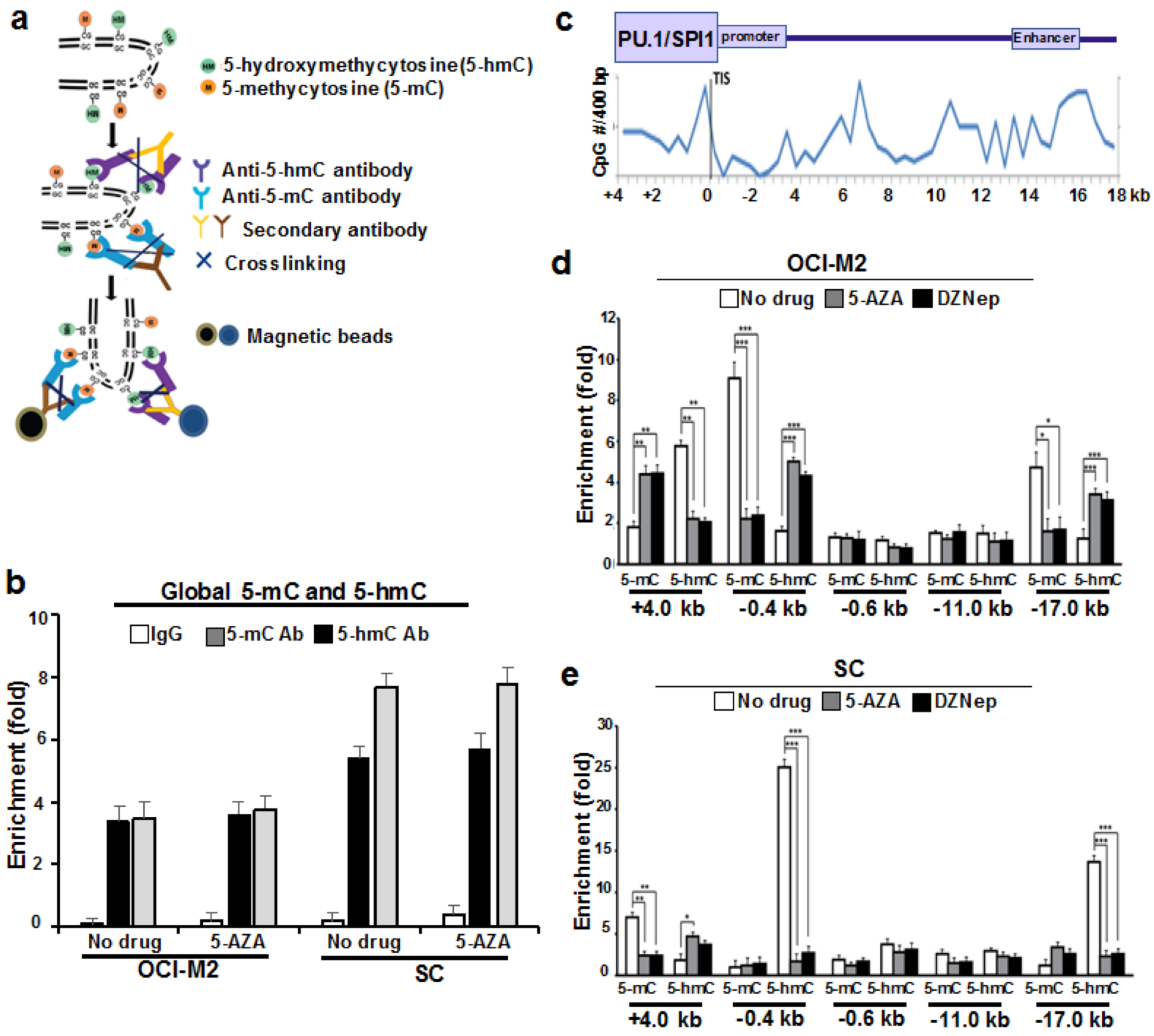

Supplementary Figure 2. Detection of drug-induced changes in DNA 5-methylcytosine (m^5C or 5-mC) and 5-hydroxyomethlcytosine (hm^5C or 5-hmC) in OCI-M2 and SC leukaemia cells. (a) A schematic illustration of crosslink-assisted DNA modification immunoprecipitation assay (CDMIA), a modified methylated DNA immunoprecipitation (MeDIP). **(b)** Measurements of the global levels of 5-mC and 5-hmC of DNA by ligation-mediated PCR of the MeDIP samples from OCI-M2 and SC cells with no drug treatment and 5-AZA treatment (1 μM for 3 hrs). **(c)** Distribution of the number of CpG dinucleotides in the entire (18 kb) upstream regulatory region and the 4-kb region downstream of the transcription initiation site (TIS) of *SPI1/PU.1*. **(d)** Measurements of the local levels of 5-mC and 5-hmC of DNA by MeDIP in the OCI-M2 cells with no drug treatment, 1 μM 5-AZA and 1 μM DZNep for 3 hrs. **(e)** Measurements of the local levels of 5-mC and 5-hmC by MeDIP in the SC cells with no drug treatment, 1 μM 5-AZA and 1 μM DZNep for 3 hrs.

Data are represented as the mean ± SEM of n = 3 independent samples. **P* < 0.05, ***P* < 0.01 and ****P* < 0.001 by Student's t-test.

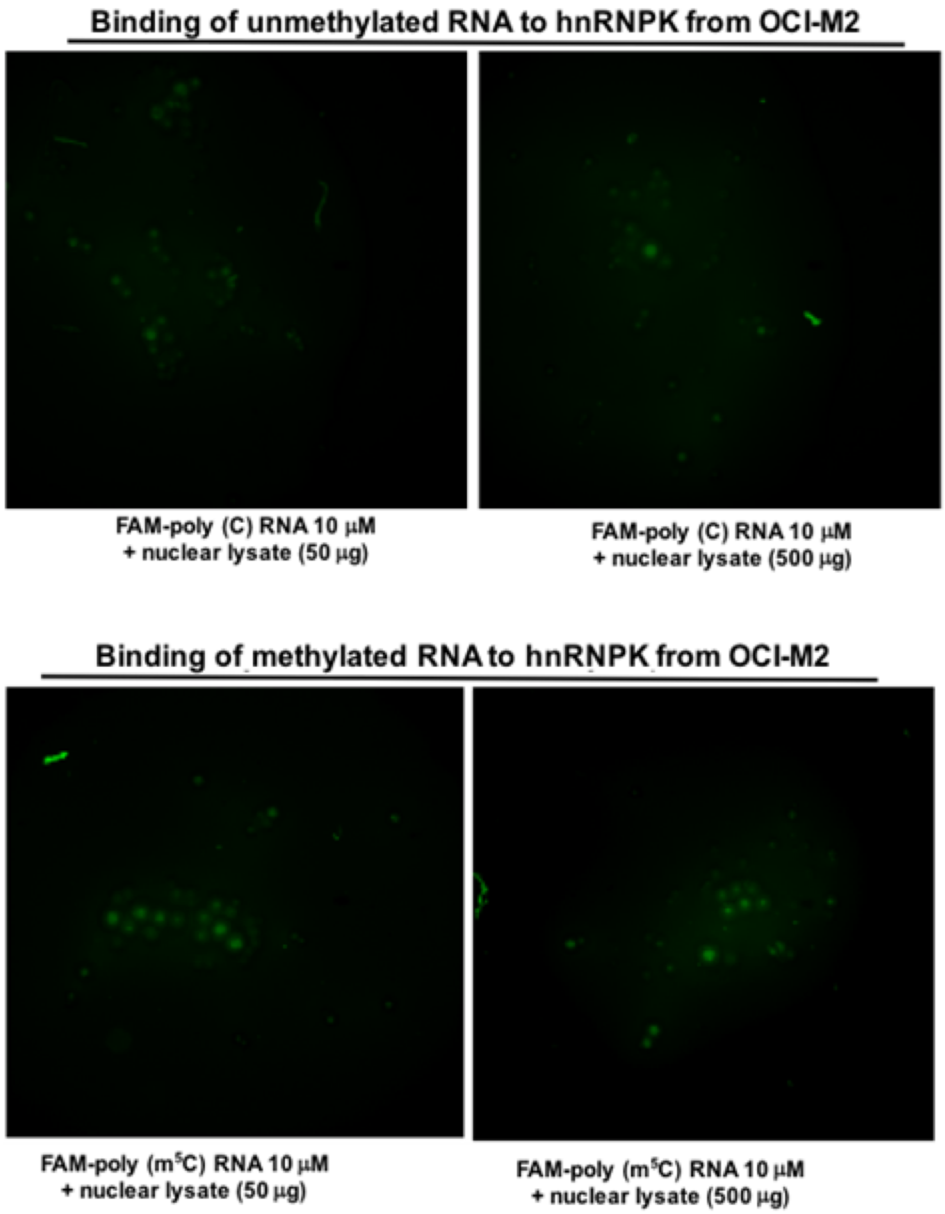

Supplementary Figure 3. Visualization and measurement of the binding of endogenous hnRNPK to the unmethylated and cytosine-methylated fluorescein (FAM)-labeled RNA oligos using nuclear lysate isolated from OCI-M2 cells.

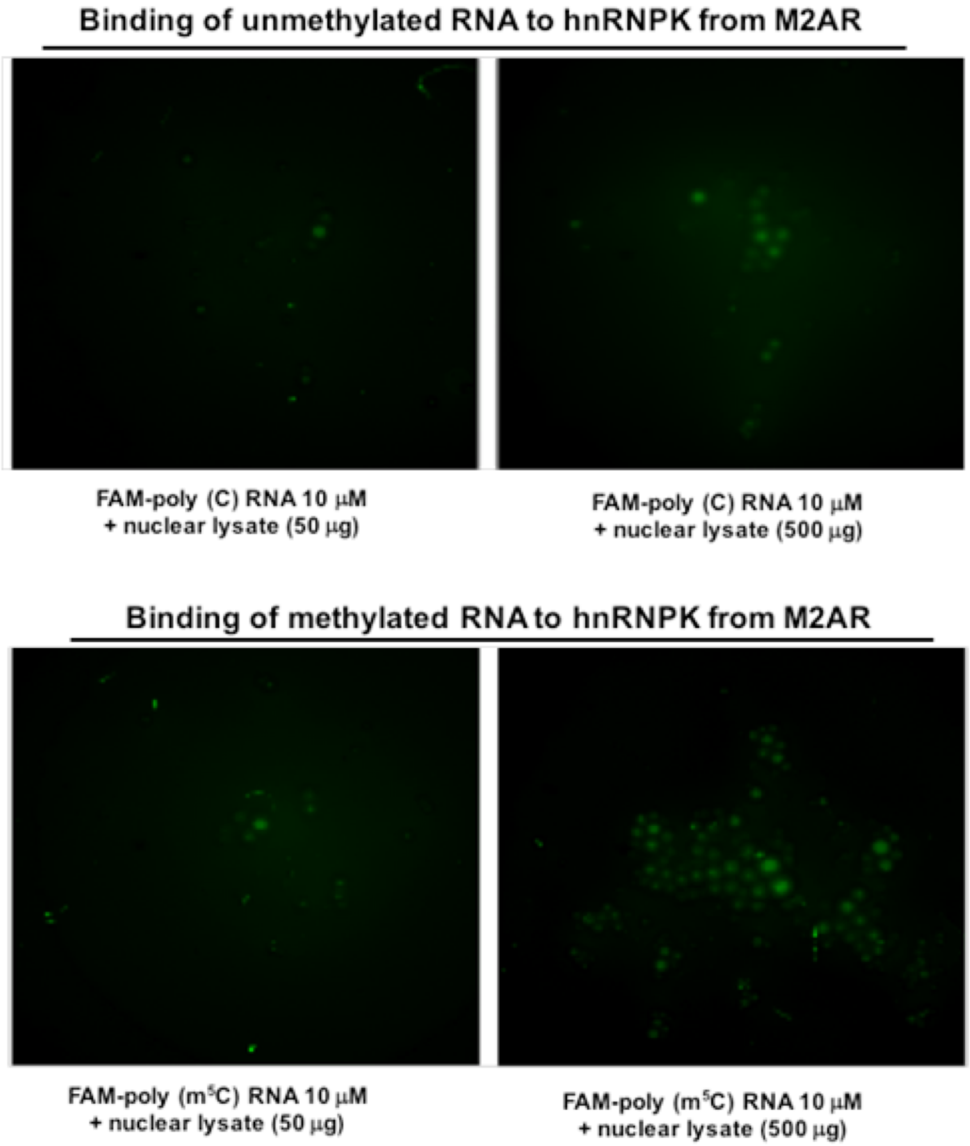

Supplementary Figure 4. Visualization and measurement of the binding of endogenous hnRNPK to the unmethylated and cytosine-methylated FAM-labeled RNA oligos using nuclear lysate isolated from M2AR cells.

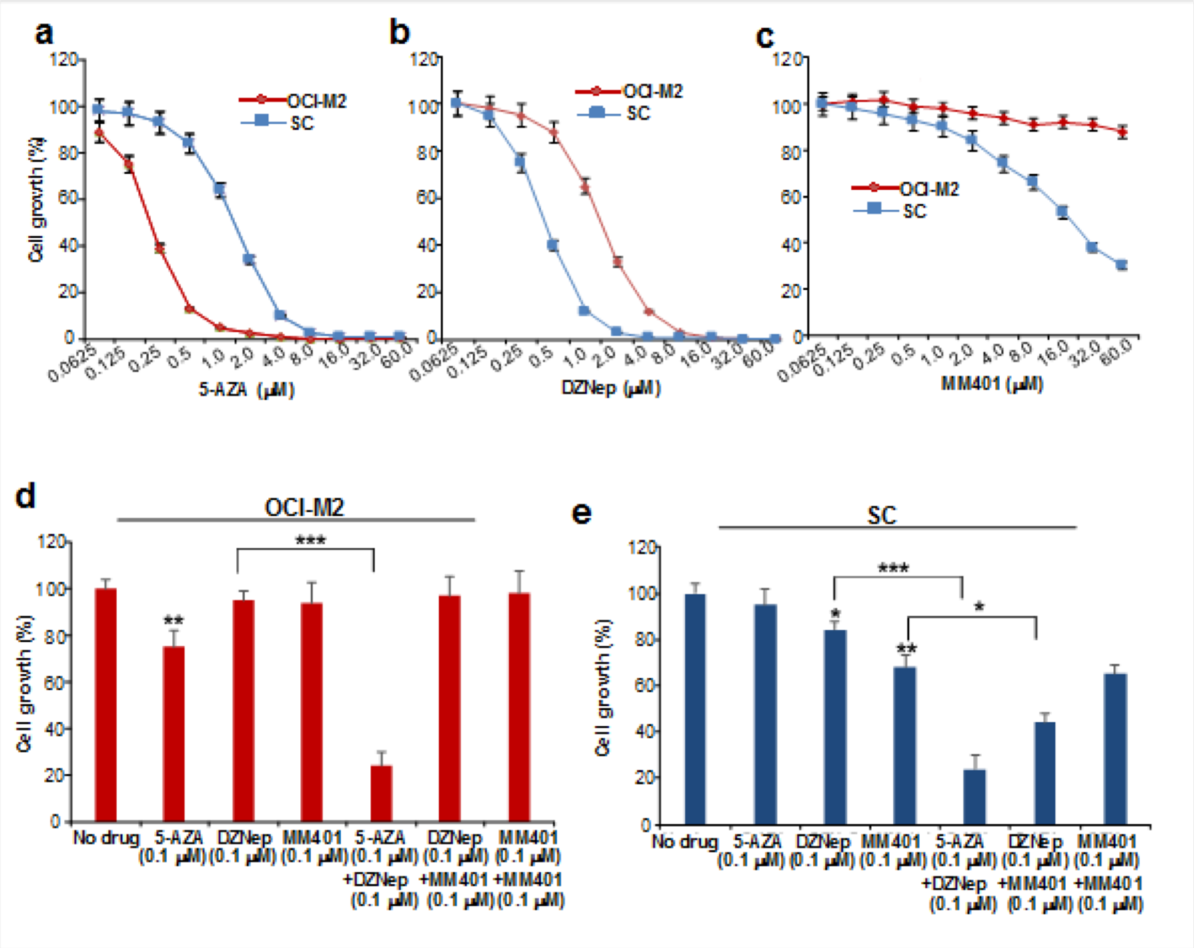

Supplementary Figure 5. Examination of the effects of various epigenetic drugs on the growth of OCI-M2 and SC cells. (a) Measurements of the growth of OCI-M2 and SC cells treated with 5-AZA for 3 days. **(b)** Measurements of the growth of OCI-M2 and SC cells treated with 3-Deazaneplanocin A (DZNep), a histone methyltransferase EZH2 inhibitor, for 3 days. **(c)** Measurements of the growth of OCI-M2 and SC cells treated with MM-401, a MLL1 H3K4 methyltransferase inhibitor, for 3 days. **(d)** Measurements of the growth of OCI-M2 cells treated with combinations of these drugs for 3 days. **(e)** Measurements of the growth of SC cells treated with combinations of these drugs for 3 days.

Data are represented as the mean ± SEM of n = 3 independent samples. *$P < 0.05$, **$P < 0.01$ and ***$P < 0.001$ by Student's t-test.

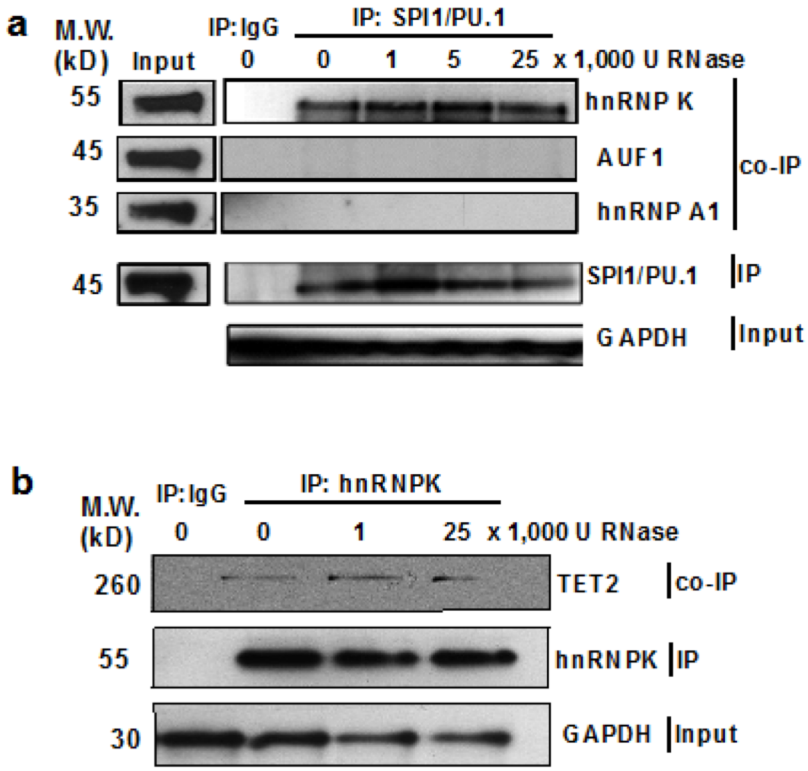

Supplementary Figure 6. Identification of hnRNPK as a direct binder of both GATA1 and TET2. **(a)** IP with anti-SPI1/PU.1 antibody and co-IPs with antibodies against various hnRNPs, such as hnRNPA1 and AUF1, in SC cells. (b) IP with anti-hnRNPK antibody and co-IP with anti-TET2 antibody in SC cells.

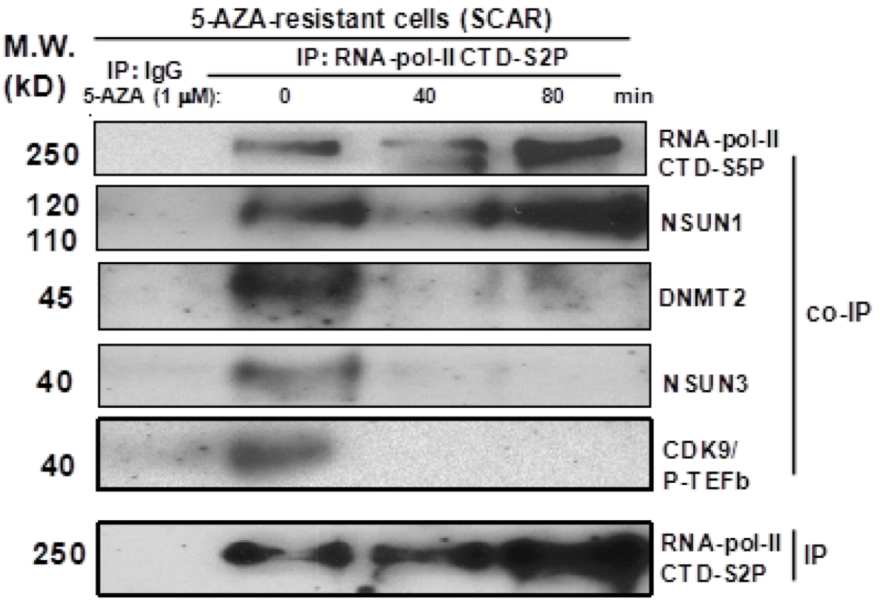

Supplementary Figure 7. Examination of the effects of 5-AZA on the interactions between RNA-pol-II CTD-S2P and RCMTs and active RNA-pol-II components in the 5-AZA-resistant SCAR cells by IP and co-IP.

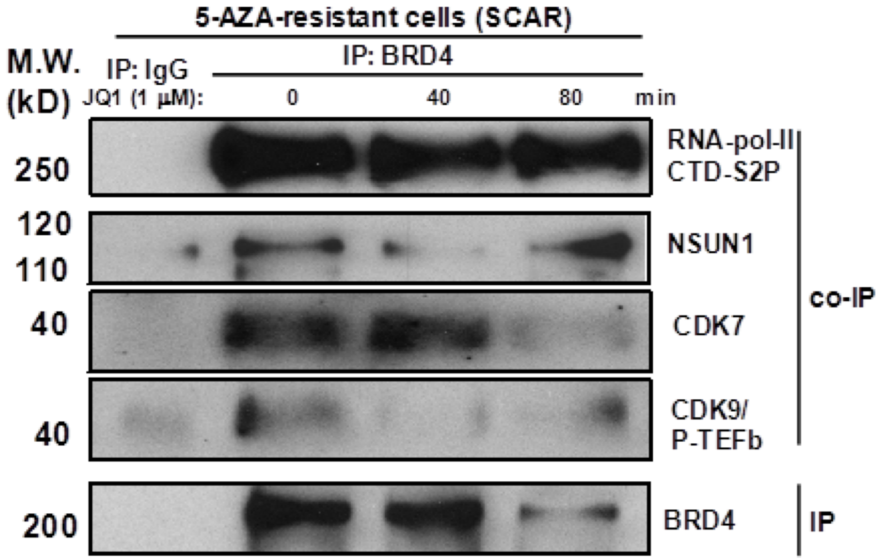

Supplementary Figure 8. Examination of the effects of JQ-1 on the interactions between BRD4 and RNA-pol-II CTD-S2P as well as NSUN1 and active RNA-pol-II components in the 5-AZA-resistant SCAR cells by IP and co-IP.

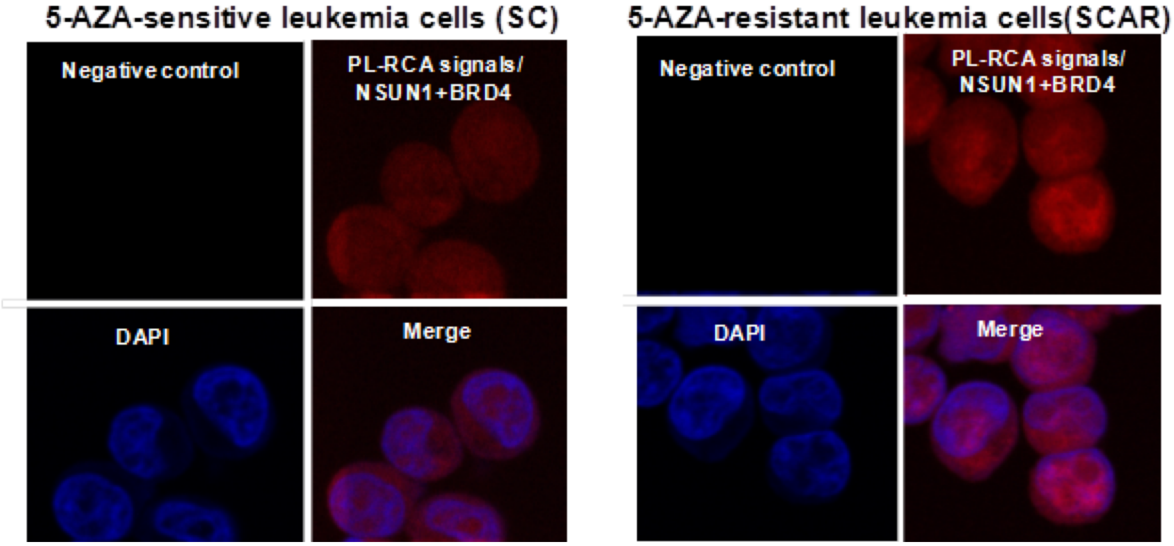

Supplementary Figure 9. Examination of co-localization of NSUN1 and BRD4 by PL-RCA in SC and SCAR cells.

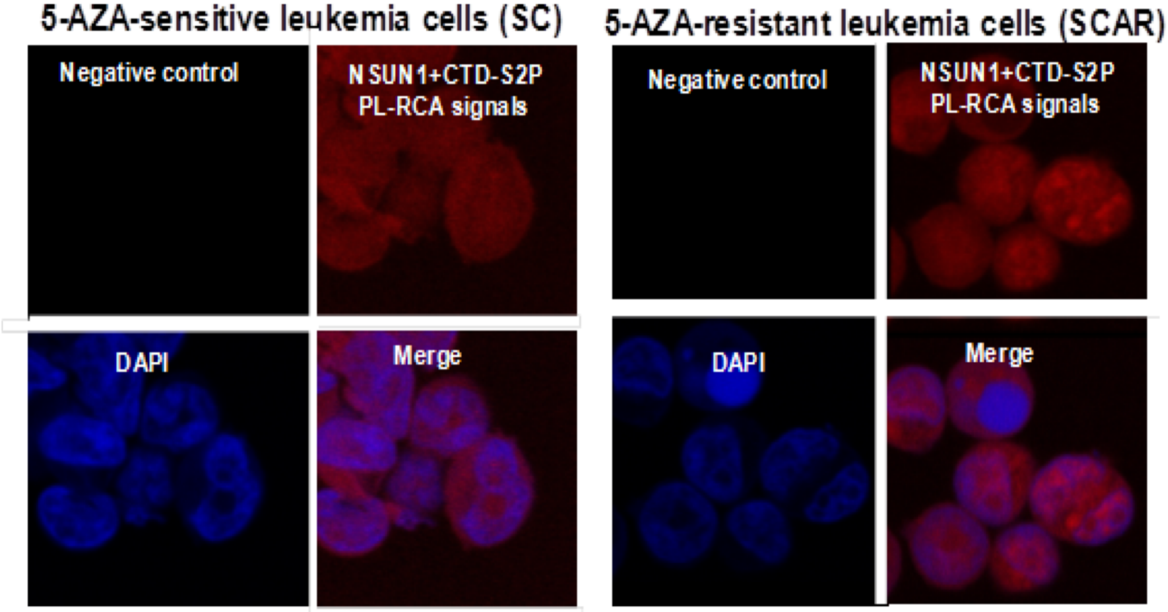

Supplementary Figure 10. Examination of co-localization of NSUN1 and RNA-pol-II CTD-S2P by PL-RCA in SC and SCAR cells.

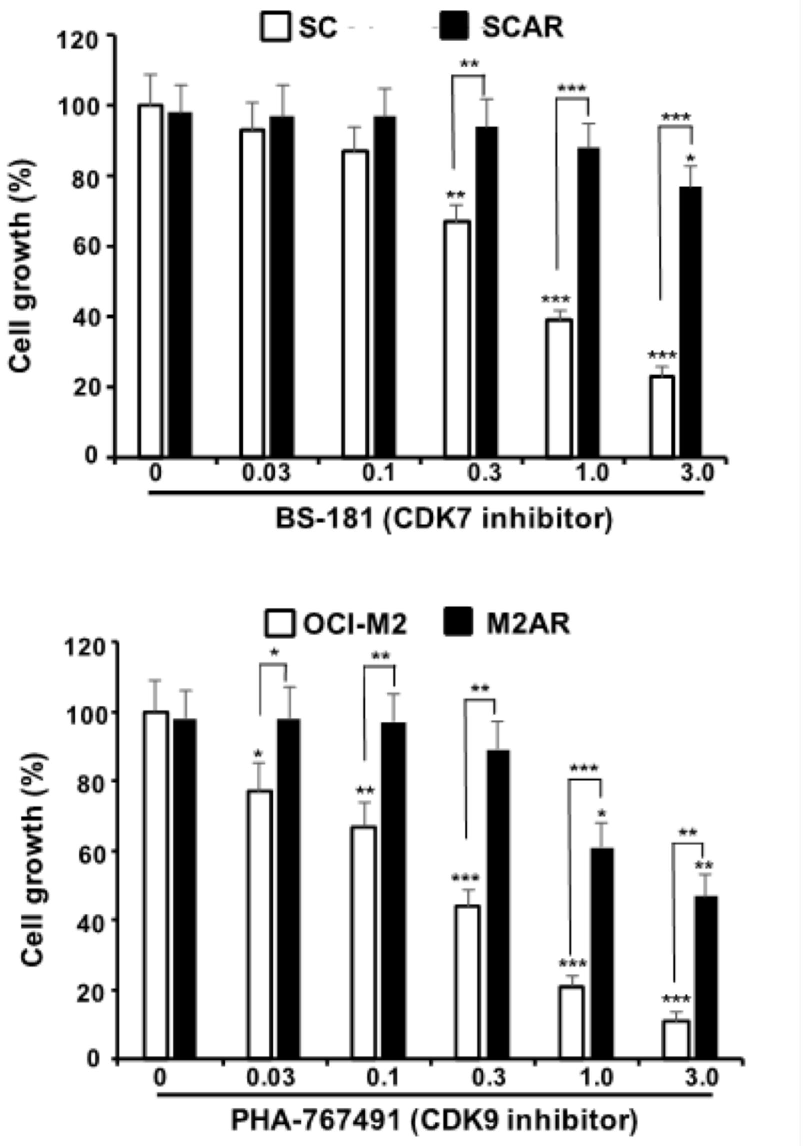

Supplementary Figure 11. Measurements of the cell growth rates in SC and SCAR cells treated with the CDK7 inhibitor BS-181 and the CDK9 inhibitor PHA-767491 at various concentrations for three days.

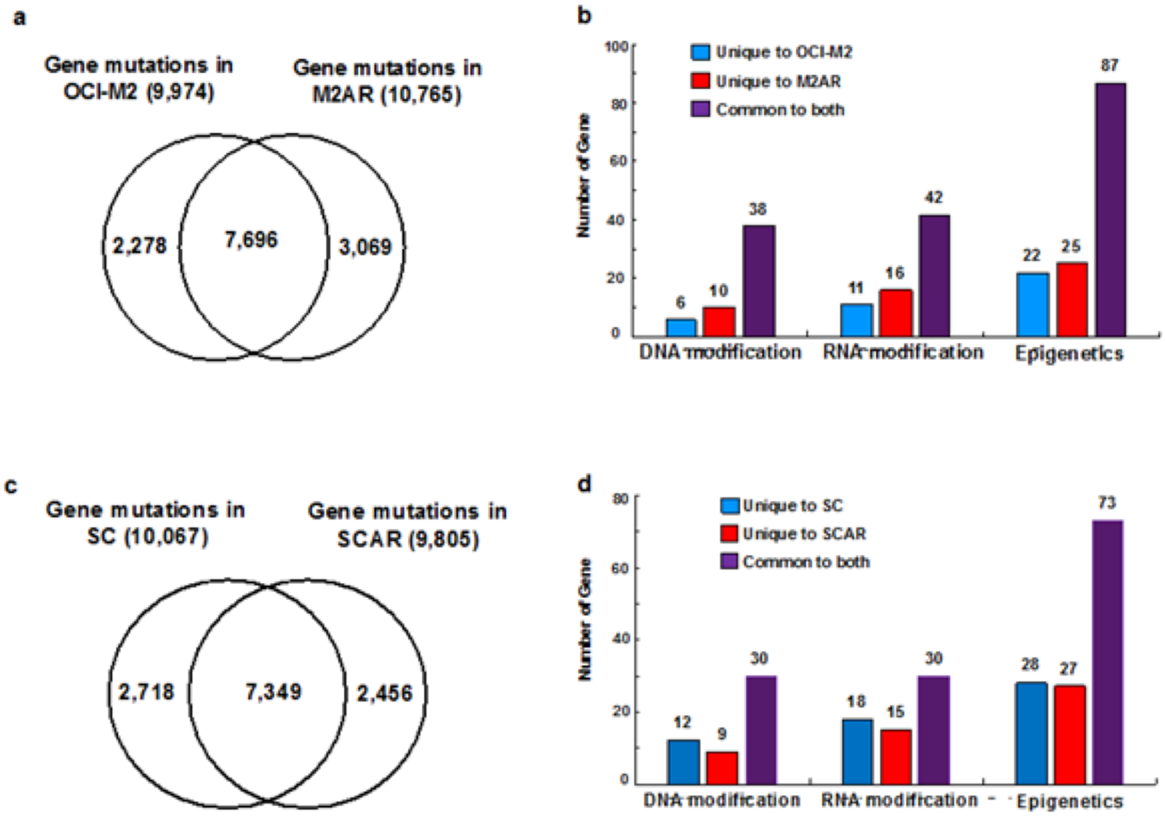

Supplementary Figure 12. Identification and ontology analysis of the gene mutations identified in the 5-AZA-sensitive OCI-M2 and SC leukaemia cell lines as well as the 5-AZA-resistant leukaemia M2AR and SCAR cell lines. (a) Venn diagram depicting the distribution of gene mutations affected in OCI-M2 and M2AR. **(b)** Graphical depiction of distribution of the gene mutations involved in the DNA modification, RNA modification and Epigenetic regulation of gene expression in OCI-M2 and M2AR. **(c)** Venn diagram depicting the distribution of gene mutations affected in SC and SCAR. **(d)** Graphical depiction of distribution of the gene mutations involved in the DNA modification, RNA modification and Epigenetic regulation of gene expression in SC and SCAR.

SUPPLEMENTARY TABLES

Supplementary Table 1. Lineage/differentiation, cytogenetics, proliferation rates and drug sensitivities of myeloid leukemia cell lines used in this study.

Cell lines	Lineages/ differentiation	Recurrent cytogenetic abnormalities (RCA)	Doubling time (hrs)	IC50 (concentrations) , M			
				5-AZA	DZNep	MI-2	MM-401
OCI-M2	Erythroid	No RCA, +6, +8, +20, -21	48	0.3	1.8	>30	>60
K562	Erythroid/ CML blast phase	t(9;22)(q34.1;q11.2) BCR-ABL1	24	0.4	1.0	>30	>60
MonoMac6	Monocytic	t(9;11)(p21.3;q23.3) KMT2A-MLLT3	36	3.0	0.2	2.5	14.0
SC	Monocytic	No RCA	26	1.6	0.3	3.8	12.0
THP1	Monocytic	t(9;11)(p21.3;q23.3) KMT2A-MLLT3	25	2.5	0.7	4.0	21.0
U937	Monocytic	t(10;11)(p13;q14) PICALM-MLLT10	24	3.8	1.2	6.8	26.8
MV4-11	Biophenotypic B/myeloid	t(4;11)(q21.3;q23.3) KMT2A-AF4; FLT3-ITD	22	2.0	1.8	4.5	12.8
HL-60	Granulocytic	-5, -17p	28	3.0	2.0	4.8	28.o
KASUMI-1	Granulocytic	t(8;21)(q22;q22.1) RUNX1-RUNX1T1	40	3.0	4.2	12.0	32.0
NB4	Promyelocytic	t(15;17)(q24.1;q21.2) PML-RARA	34	5.0	4.0	24.0	40.0

5-AZA (5-Aacytidine, 5-aza-2'-deoxycytidine), hypomethylating agent; DZNep (3-Deazaneplanocin A), an S-adenosyl-L homocysteine hydrolase inhibitor which induces EZH2 protein depletion; MI-2 and MM401, MLL inhibitors.

Supplementary Table 2. Clinical specimens used in this study.

Case ID	Diagnosis	Blast count	Immunophenotype	Cytogenetics	Gene mutations
AS-1	AML (M5)	81%	CD34-, MPO+, CD117+, CD33+, CD11b+, CD7+	Normal	*NPM1*
AS-2	t-AML	83%	CD34-, CD117-, CD33+, CD11b+, MPO+,	47,XY,+11	*DNMT3A, TET2*
AS-3	t-AML	31%	CD34-, CD117+, CD33+, CD11b+, MPO+, CD4+	ND	*ND*
AS-4	AML-MRC	36%	CD34+, CD117+, CD33+, CD11b+, CD7+, MPO+	46,XY,der(17)t(1;17)(q21:p13.1)	*DNMT3A, P53*
AS-5	AML-MRC	51%	CD34+, CD117+, CD33+, MPO+, CD5+, CD11b+,	Complex	*TET2, SXL1, SRSF2,*
AS-6	MDS/RAEB-2	15%	CD34+, CD117+, CD33+, CD11b-, MPO+	Normal	ND
AS-7	AML (M5)	70%	D34+, CD117+, CD33+, MPO+, CD4+, CD11b+,	Normal	No recurrent mutations
AS-8	AML-MRC	90%	CD34+, CD117+, CD33+, MPO+, CD7+	Complex/loss of 5q, +1, +14,+17	TP53, TET2
AS-9	MDS/RAEB-1	6%	C34+, CD117+, MPO+, CD33+	Normal	ND
AR-1	AML-(M5)	48%	D34-, MPO+, CD117+, CD33+, CD11b+	Normal	NPM1, RAS
AR-2	t-AML	27%	CD34+, CD33+, MPO+, CD11b+	46,XY,t(6;11)(q27;q23.3)	NRAS
AR-3	AML-MRC	30%	CD34+/-, MPO-, CD117+, CD33+, CD235a+	Complex/loss of 5q, -7, +8	TP53, TET2
AR4	AML (M5)	33%	D34+, CD117+, CD11b+, MPO+, CD7+, CD4+	t(3;18)(p22;p11.2)	NPM1
AR5	AML (M5)	26%	CD34+, CD117+, CD11b+, MPO+, CD7+	Normal	No recurrent mutations
AR6	MDS/RAEB-2	17%	CD34+, CD117+, CD33+, CD11b-, CD7+	Normal	ND
AR7	AML (M5)	56%	CD34-, CD117-, CD33+, CD11b+, MPO+	47,XY, +11,t(X;18)	ND
AS-8	MDS/RAEB-1	8%	C34+, CD117+, MPO+, CD33+	Normal	ND
AR-9	AML (M4)	85%	CD34+, CD117+, CD33+, MPO+	Normal	TET2, ASXL1, SRSF2,

AS, 5-AZA-sensitive; AR, 5-AZA-resistant; ND, not done; t-AML, therapy-related AML; AML-MRC, AML with myelodysplasia-related changes.

Supplementary Table 3. Reagents used in this study and their resources.

Experimental reagents	Sources and catalog numbers
5-azacytidine (5-AZA), 5-fluorocytidine (5-FU), 2'-deoxy-5-azacytidine (Decitabine, DC), 2'-deoxy-5-fluorocytidine (5-FDOC)	Sigma-Aldrich; Catalog numbers: A2385, 543020, A3656, T159638, respectively
3-deazaneplanocin A (DZNep)	Cayman Chemical company/G6423
MM401	Provided by Yali Dou (Cao et al. Mol Cell. 2014 53; 247)
5-hydroxymethylcytosine (5-hmC) antibody, 5-methylcytosine (5-mC) antibody, TET1 antibody, TET2 antibody, TET3 antibody	ActiveMotif; Catalog numbers: 39769, 39649, 61443, 61389, 61395, respectively
DNMT1 antibody, RNA-pol-II (CTD total) antibody, RNA-pol-II (CTD S2P) antibody, RNA-pol-II (CTD S2P/S5P) antibody, RNA-pol-II (CTD S5P) antibody, EZH2 antibody, GAPDH antibody, U2AF1 antibody, AUF1/hnRNPD antibody, hnRNPA1 antibody, CDK7 antibody, CDK9 antibody, BRD2 antibody, BRD4 antibody	Cell Signaling: Catalog numbers: 5032, 2629, 13499, 13546, 13523, 13705, 5246, 2118, 12382, 8433, 2916, 2316, 5848, 13440, respectively
DNMT2 antibody, NSUN3 antibody	Sigma-Aldrich; Catalog numbers: WH0001787M1, AV48884, respectively
DNMT3A antibody, DNMT3B antibody	Invitrogen; Catalog numbers: PAS-11159, PA3-16558, respectively
EZH1 antibody	EMD Millipore; Catalog number: ABE281
GATA1 antibody, PU.1 antibody, hnRNPK antibody, CD34 antibody	Thermo Fisher Scientific; Catalog numbers: PA1-099, MA1-087, 11-0349-42, respectively
NSUN1 antibody, NSUN2 antibody	Santa Cruz; Catalog number: SC-398884 and SC-366094, respective
hnRNPK recombinant protein	LifeSpan Bioscience; Catalog number: LS-G21413
FAM-poly(c) RNA oligo, FAM-poly(m^5C) RNA oligo	Integrated DNA Technologies
Click-iT RNA imaging kits	Invitrogen; Catalog number: C10329
Duolink PLA kit	Sigma-Aldrich; Catalog number: DUO92101
BS-181, PHA-767491, JQ1	Selleck Chemicals; Catalog numbers: S1572, S2742, S7110, respectively
MTT cell proliferation kit	ATCC; Catalog number: 30-1010k

Supplementary Table 4. The sequences of the primers for Crosslink-assistant DNA Modification Immunoprecipitation (CDMIP), 3C and RNA-pol-II ChIP experiments in this study.

Primer Names	Positions at *SPI1/PU.1*	Primer sequences (5' to 3')
**SPI1+4k-L**	+3.9 kb (downstream of TSS)	ttcaggagtgacaccttcctagg
**SPI1+4k-R**	+4.1 kb (downstream of TSS)	gacccaaagcgaggtggcctagtc
**SPI1-1L**	-0.46 kb (upstream of TSS)	aggaagaaagcagcactatgctg
**SPI1-1R**	-0.37 kb (upstream of TSS)	ctttctggagtcagccttcctgg
**SPI1-2L**	-4.1 kb (upstream of TSS)	gcttaagttgattctcagccag
**SPI1-2R**	-3.9 kb (upstream of TSS)	tacacttggtctcaaaggtgca
**SPI1-3L**	-9.8 kb (upstream of TSS)	gagcctctcccagtgtctcaa
**SPI1-3R**	-9.6 kb (upstream of TSS)	gagcctctcccagtgtctcaa
**SPI1-11k-L**	-10.9 kb (upstream of TSS)	gcctcggaggtttgggcttcag
**SPI1-11K-R**	-10.75 kb (upstream of TSS)	cctgtgcctccgtggctgtccct
**SPI1-4L**	-15.1 kb (upstream of TSS)	ctgttccctgccttggtgtttca
**SPI1-4R**	-14.95 kb (upstream of TSS)	ttgcaacctgtggtcactgagg

TSS, transcription start site

Supplementary Table 5. Sequences of siRNAs used in this study and their resources

Gene name	Sequence (5' to 3')	Sources/ cat. No.
HNRNPK	A: rCrGrArUrGrArArArCrCrUrArUrGrArUrUrArUrGrGrUrGGT<br>B: rCrCrArArCrArCrUrArUrArArArGrGrArArGrUrGrArCrUTT<br>C: rArGrUrArCrUrArCrArArGrUrUrGrArGrUrArArUrGrGrUAT	OriGene/ SR302173
NSUN1	A: rCrUrGrGrArCrUrArGrUrGrGrUrGrUrArUrGrATT<br>B: rGrUrGrUrGrArUrCrCrUrUrGrCrCrArArUrGrATT<br>C: rGrGrUrArGrArCrUrArUrGrCrUrCrUrGrArArATT	Santa Cruz/SC-75962
NSUN2	A: rGrGrUrGrUrArGrArArArUrArArCrArGrCrGrGrUrGrArAGA<br>B: rArGrArUrGrUrUrArArGrArUrArCrUrGrUrUrGrArCrCrCAG<br>C: rArGrArArUrGrArArCrGrGrCrUrUrCrArUrUrArUrCrUrCAG	OriGene/ SR310319
NSUN3	A: rCrUrArCrArGrArUrArGrArGrCrUrGrUrUrArATT<br>B: rCrUrCrUrGrGrGrUrCrUrGrUrUrUrGrGrArArUTT<br>C: rCrUrCrUrGrGrGrUrCrUrGrUrUrUrGrGrArArUTT	Santa Cruz/SC-78202
DNMT2 (TRDMT1)	A: rGrGrUrUrGrArGrArArUrArUrCrUrArCrArArArUrCrCrCTT<br>B: rGrGrArArUrGrUrArGrCrArUrGrArCrGrUrUrArArGrArUTT<br>C: rGrCrArArCrArUrArCrArCrUrCrUrCrArArUrGrArArCrUTT	OriGene/ SR301245
BRD4	A: rCrUrGrArArCrCrUrCrCrCrUrGrArUrUrArCrUTT<br>B: rCrCrArArCrUrGrCrUrArCrArArGrUrArCrArATT<br>C: rCrArGrCrUrArArGrUrCrUrArGrArUrArUrCrATT	Santa Cruz/SC-141740
